# Analysis of the Chemical Composition, Antimicrobial, and Antioxidant Qualities of Microwave and Supercritical CO_2_-Extracted Lavender Essential Oils Cultivated in a Hyperarid Region of Türkiye

**DOI:** 10.3390/molecules29235605

**Published:** 2024-11-27

**Authors:** Ayça Gedikoğlu, Hale İnci Öztürk, Afranur Özçoban

**Affiliations:** 1 Department of Food Engineering, Faculty of Engineering and Architecture, Konya Food and Agriculture University, Konya 42080, Türkiye; 2 Department of Food Engineering, Faculty of Chemical Metallurgical Engineering, Yıldız Technical University, Istanbul 34210, Türkiye; haleinciozturk@hotmail.com

**Keywords:** GC-MS, bioactivity, microbial inhibition, extraction techniques, volatile components

## Abstract

This study compares the chemical composition, antioxidant capacity, and antibacterial properties of lavender essential oils extracted using microwave-assisted extraction (MAE) and supercritical CO_2_ extraction (SCDE). Gas chromatography–mass spectrometry analysis revealed that the MAE oil contained higher levels of linalyl acetate (36.19%) and linalool (28.29%) compared with the SCDE oil, which had values of 28.72% and 27.48%, respectively. The MAE oil also showed superior antioxidant activity, with DPPH IC_50_ values of 72.99 mg/mL and FRAP values of 1.31 mM Fe^2+^/g, compared with 80.84 mg/mL and 1.14 mM Fe^2+^/g for the SCDE oil. Antibacterial tests indicated that the MAE oil exhibited lower MIC values, demonstrating twice the antibacterial activity against *Bacillus cereus* NRRL B3711, *Bacillus subtilis* PY79, and *Enterococcus faecalis* ATCC 29212 compared with the SCDE oil. These results highlight the superior bioactivity of MAE-extracted lavender oil, making MAE a preferred method for high-quality oil extraction from drought-affected lavender plants.

## 1. Introduction

Lavender, scientifically known as *Lavandula angustifolia*, is a perennial shrub belonging to the *Lamiaceae* family, which is popular for its pleasant scent and medicinal properties [1]. Lavender oil is one of the primary essential oil crops cultivated in the Mediterranean region and Türkiye [2]. It is widely used in pharmaceutical and cosmetic industries due to its various beneficial effects, such as antioxidant, antiseptic, analgesic, antispasmodic, antihypertensive, healing, and anti-inflammatory properties. In the food sector, lavender is primarily added to beverages, baked goods, chocolate, ice cream, and chewing gum, among other products, for its aromatic qualities [3,4]. In addition to these properties, lavender oil has shown antimicrobial activity against a wide range of micro-organisms in in vitro studies [5,6] and in situ experiments [7,8], indicating its potential as a preservative for the food industry.

Previous research has revealed that the bioactivity of essential oils is linked to the existence of terpenes, sesquiterpenes, oxygenated terpenes, aldehydes, and other compounds [9,10]. Various studies have shown that lavender oils possess antibacterial properties against different foodborne pathogens due to the presence of active elements, such as linalool, linalyl acetate, camphor, caryophyllene, beta-phellandrene, and eucalyptol [11,12,13]. The levels and types of these active components can be influenced by factors such as geographical location, environmental factors, and processing methods [4,5,9]. Drought conditions are known to impact the bioactive elements in essential oils, with one study revealing that the presence of key compounds decreased initially under drought stress but increased with recurring stress [14]. Another study demonstrated that the yield of essential oil under drought conditions depends on the raw weight and species of the plant [15]. A recent study by Mansinhos et al. [16] found that, under extreme heat and salt conditions, the essential oil of *L. viridis* possessed higher quantities of 1,8-cineole and camphor. They also observed that the essential oil’s enzyme and antioxidant activities increased in line with the changes in chemical composition.

An additional factor influencing the concentration of active compounds in essential oils is the extraction methods employed. Traditionally, lavender essential oil has been obtained through steam distillation or hydro-distillation processes. However, these processes involve extended exposure to high temperatures, which can lead to the loss of some volatile compounds. To address this issue, modern techniques such as microwave and supercritical fluid extraction have been adopted to increase processing efficiency and minimize the loss of active compounds [17]. In a recent study, traditional distillation and microwave extraction methods were compared to assess the antimicrobial and antioxidant potential of common and black thyme [9]. The results showed that microwave-extracted essential oils of common and black thyme exhibited higher bioactivity. Similarly, it was reported that the microwave hydro-diffusion method provided the highest yield with the most desirable aroma profile [18]. In contrast, a separate study found no significant variations in chemical composition and oil production between hydro-distillation and supercritical carbon dioxide extraction methods for the essential oils of some *Lamiaceae* plants. The authors also emphasized that the effectiveness of extraction varied depending on the plant material, indicating that supercritical carbon dioxide extraction was the most effective method for extracting savory oils [19]. Most of the earlier research demonstrates that extraction techniques and environmental stressors, such as heat and drought, have a significant influence on the biological activity of essential oils. There is a limited number of studies on the chemical composition and bioactivity of Turkish lavender oils from plants grown under drought conditions. In particular, Konya Province has alkaline, salty soil with major drought stress. Moreover, there is no available study to compare major green extraction technologies to assess the essential oils of lavender cultivated in Türkiye. Therefore, this study aimed to evaluate the effects of two environmentally friendly extraction techniques (supercritical carbon dioxide and microwave) on the chemical composition, antioxidant capacity, and antimicrobial qualities of lavender essential oil extracted from plants cultivated in the extremely arid region of Türkiye.

## 2. Materials and Methods

### 2.1. Materials

**Figure 1:**
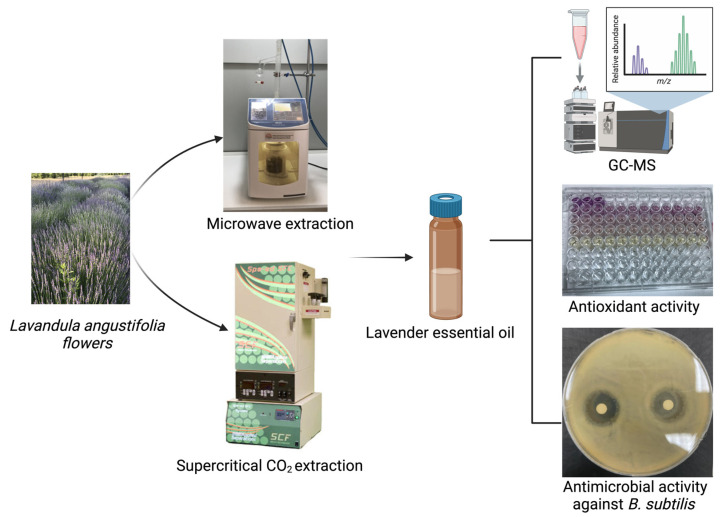
The extraction process of the lavender flowers and the in vitro analysis of the essential oil.

*Lavandula angustifolia* was grown on the campus of the Konya Food and Agriculture University (KFAU) in Konya, Türkiye. The flowers were harvested in August 2022 and dried in the laboratory on open shelves at ambient temperatures. Once the flowers were completely dried, they were picked and passed through multiple shifters to remove soil and other debris using a Retsch (Haan, Germany) AS 200 Digit CA sieve shaker. Finally, lavender samples were placed in airtight containers and maintained in a dark room at room temperature before further analysis. Figure 1 displays the extraction processes of lavender flowers and the in vitro analysis of the essential oil.

Chemicals were purchased from Sigma-Aldrich (Steinheim, Germany). Merck KgaA (Darmstadt, Germany) supplied the peptone water, tryptic soy broth, and Mueller–Hinton agar. Empty sterile test discs were purchased from Oxoid (Basingstoke, UK). Bacterial cultures of *Bacillus* (*B.*) *cereus* NRRL B3711, *B. subtilis* PY79, *Enterococcus* (*E.*) *facealis* ATCC 29212, *Listeria* (*L.*) *monocytogenes* ATCC 19115, *Staphylococcus* (*S.*) *aureus* ATCC 9144, (*S.*) *epidermidis* ATCC 12228, *Escherichia* (*E.*) *coli* ATCC 25922, *Salmonella* (*S.*) Enteritidis ATCC 13076, and *S.* Typhimurium ATCC 14028 were provided by the microbiology laboratory of KFAU.

### 2.2. Microwave-Assisted Extraction (MAE)

The method of Liu et al. [1] was used with slight modifications for the MAE. The lavender flowers were first ground into a fine powder, and then fifty grams of ground lavender was allowed to steep in distilled water (1:4, *w/v*) for 15 min. Afterward, using NEOS microwave extraction equipment (MA 125 Milestone, Milan, Italy), the extraction was conducted at 500 W for 40 min until no more essential oil was recovered. Subsequently, any water was removed from the essential oil by gathering it and placing it in a vial with anhydrous sodium sulfate. The vials were then kept for further investigation at 4 °C.

### 2.3. Supercritical CO_2_ Extraction (SCDE)

In this analysis, a high-pressure, continuous-flow supercritical fluid extraction (SFE) system (Applied Separations Speed SFE-2, Allentown, PA, USA) was employed. To conduct the SCDE, the methodology of Danh et al. [20] was adapted with minor changes. First, 50 g of milled lavender flowers was placed into an SFE vessel containing glass wool, equipped with a compressor (Atlas Copco GX-4FF, Wilrijk, Belgium). Then, lavender essential oil was extracted by using supercritical carbon dioxide at a flow rate of 1.5 mL/min with a density of 0.9 g/mL. The supercritical carbon dioxide extraction system operated at a pressure of 150 bar and a temperature of 50 °C.

### 2.4. Yield (%)

The yield of the essential oil was calculated according to the following formulation (Equation (1)):

(1) $$Y\;(\%)=\frac{\mathrm{Weight}\;\mathrm{of}\;\mathrm{essential}\;\mathrm{oil}}{\mathrm{Weight}\;\mathrm{of}\;\mathrm{plant}\;\mathrm{material}}\;\times \;100$$

### 2.5. Gas Chromatography–Mass Spectrometry (GC–MS)

The chemical components of lavender essential oils extracted using supercritical carbon dioxide and microwave treatment were evaluated using GC-MS, utilizing the previously reported methodology [9]. The GC-MS conditions are summarized in Table 1. Briefly, a GC-MS QP2020 fitted with a Rxi-5Sil MS column (5% diphenyl-95% dimethylpolysiloxane 30 m × 0.25 mm i.d., df = 0.25 μm; RESTEK GC Columns, Centre County, PA, USA) was used to analyze the essential oil. The temperatures of the injector and detector were fixed at 250 °C. The oven’s temperature was programmed to start at 40 °C for 2 min and increase to 240 °C at a rate of 4 °C per minute and then maintain the temperature for 53 min. The carrier gas was helium, with a linear velocity of 43.4 cm/s. Following an n-hexane (1:10, *v/v*) dilution of the samples, 1.0 μL was injected into the GC using a split-mode injector (split ratio: 1:25). The mass range *m/z* (mass-to-charge ratio) was 40–400 amu (atomic mass units), while the ionization voltage was 70 eV (electron volts). The separated components were matched using WILEY 7, data from the National Institute of Standards and Technology mass spectrum collection, and authentic standards. The proportion of combinations was calculated using peak area integration. This analysis was carried out in duplicate.

### 2.6. DPPH Free-Radical Scavenging Activity

An earlier established method [21], with slight modifications, was used to measure the 2,2-diphenyl-1-picrylhydrazyl (DPPH) free-radical scavenging activity of lavender essential oils obtained using MAE and SCDE. Initially, 5 mL of a methanolic solution containing 0.004% (*w:v*) DPPH was mixed with different concentrations of lavender essential oils (9.5, 18.8, 27.9, and 54.4 mg/mL). Following a 30 min reaction at room temperature, the absorbance at 517 nm was measured in comparison with a blank. The following formula (Equation (2)) was used to obtain the inhibition (%) of the DPPH radicals:

(2) $$I\;(\%)=\frac{{(A}_{\mathrm{blank}}-A_{\mathrm{sample}})}{A_{\mathrm{blank}}}\;\times \;100$$

In the formula, A_blank_ means absorbance of the DPPH methanolic solution and A_sample_ represents the absorbance of the lavender essential oils with the DPPH methanolic solution. Then, the oil concentrations were graphed against the inhibition percentages (%) to calculate the amount of oil (mg/mL) that provides 50% inhibition (IC_50_) using the equation derived from the graph.

### 2.7. FRAP Assay

The effect of MAE and SCDE on the ferric-reducing antioxidant power (FRAP) of lavender essential oil was determined following the method of Riahi et al. [18] with slight modifications. First, 10 mM 2,4,6-Tris(2-pyridyl-s-triazine) (TPTZ) solution in 40 mM HCl, 300 mM of acetate buffer (pH 3.6), and 20 mM of FeCl_3_·6H_2_O were prepared and mixed together (1:10:1, *v:v:v*). Using 96-well plates, 100 μL of the FRAP reagent was placed in a well with 96 mg of lavender oil. The plate was incubated at 37 °C for 15 min. The FRAP solution was used as a blank, and the absorbance was measured at 595 nm. Subsequently, standard solutions of FeSO_4_·7H_2_O at different concentrations (0.1, 0.2, 0.4, 0.6, 0.8, and 1 mM in distilled water) were prepared, and the equation from the standard curve was utilized to calculate the FRAP antioxidant activity of the lavender oils (mM of Fe^2+^/g of oil).

### 2.8. Agar Disc Diffusion Assay

The antibacterial effects of lavender essential oils were ascertained by utilizing a disc diffusion assay, as described in a recent study [10]. Lavender oils from MAE and SCDE were tested against nine foodborne bacteria. At first, each bacterium was separately activated in tryptic soy broth and allowed to incubate overnight at 35 °C. Bacterial suspensions were then precipitated at room temperature for 10 min at 1714× *g*. After washing the pellets, bacterial cultures were diluted in a 0.9% NaCl solution (*w/v*), and a densitometer was used to achieve a final cell concentration of 0.5 McFarland (1 × 10^8^ CFU/mL). Then, 100 μL of the bacterial suspensions was spread on Mueller–Hinton agar. Next, 9.6 mg of lavender oil-impregnated discs were placed on the agar surface. The empty sterile discs were utilized as the negative control. The plates were incubated at 37 °C for up to 24 h. The zone of inhibition in millimeters was measured to evaluate the antibacterial activity, excluding the radius of the disc.

### 2.9. Broth Dilution Assay

To determine the minimum inhibitory concentration (MIC) required to inhibit the selected bacteria, the 96-well microplate method explained in a recent study was utilized [10]. Initially, 100 µL of tryptic soy broth (TSB) was dispensed into each well. Subsequently, 100 µL of essential oil was dispensed into the first column of the 96-well microplate. Using a multichannel pipette for proper mixing, half of the volume was placed in the next column. This procedure was repeated until the 10th column, with the final portion being discarded. Later, 100 µL of microbial suspension was pipetted into each well, except for the 12th column. The 11th column served as a positive control containing both broth and organisms, while the 12th column acted as a negative control with only broth. The oil concentration ranged between 0.09 and 48 mg, and the inoculum density was set at 1–2 × 10^6^ CFU/mL. The microplates were then incubated at 37 °C for 18 h. The lowest concentration of oils that showed no visible bacterial growth was used to determine the MIC values.

### 2.10. Statistical Analysis

For the investigation, three separate experimental replications were carried out, and the results are expressed as the mean ± standard deviation. Using Stata IC 14 (Stata Corp., College Station, TX, USA), a two-sample *t*-test was used to evaluate the data (*p* ≤ 0.05).

## 3. Results and Discussion

### 3.1. GC-MS Analysis

**Figure 2:**
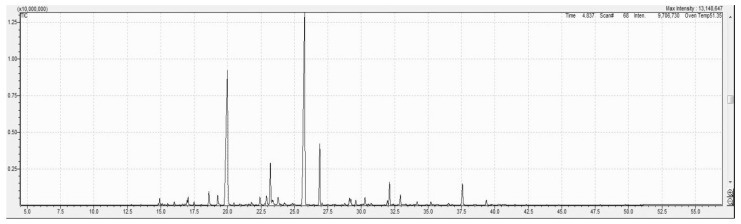
GC-MS profile of *Lavandula angustifolia* essential oil using microwave-assisted extraction.

**Figure 3:**
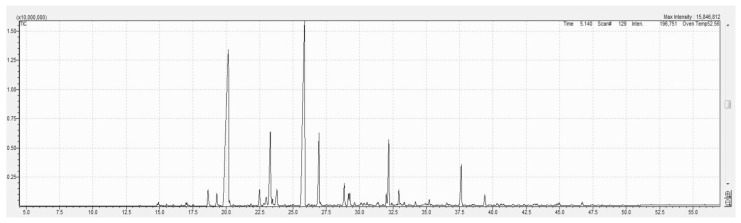
GC-MS profile of *Lavandula angustifolia* essential oil using supercritical carbon dioxide extraction.

In the MAE lavender oil (Figure 2), 70 compounds were detected, while in the SCDE lavender oil (Figure 3), 96 compounds were identified through GC-MS analysis. Only 14 compounds with a relative peak area exceeding 0.5% were evaluated for qualitative analysis, and the results are presented in Table 2. The most prevalent compounds in the lavender oils extracted using MAE and SCDE were linalool, linalyl acetate, lavandulyl acetate, 4-terpineol, and (−)-caryophyllene oxide, comprising 78.55% and 72.55% of the oil contents, respectively. For the minor components, trans-beta-caryophyllene, 1-hydroxylinalool, linalool oxide trans, and lavandulol were found in varying amounts depending on the extraction method. A significant difference was observed in the linalyl acetate content of the MAE lavender oil (36.19%), which was notably higher (*p* ≤ 0.05) than that in the SCDE lavender oil (28.72%). There was also a significant difference (*p* ≤ 0.05) in the endo-borneol content, with MAE oil having a higher concentration at 1.35%. Furthermore, alpha-terpineol (1.10%) was exclusively found in the MAE lavender oil, and 3,7-octadiene-2,6-diol, 2,6-dimethyl (1.67%) was only present in the SCDE oil.

In a recent study, Salata et al. [22] examined the effect of irrigation regime and drying method on the chemical composition of the essential oil of lavender flowers from Poland. They found that both irrigation type and drying techniques had an impact on the presence of mono- and sesquiterpenes. Moreover, in various studies, the impact of extraction techniques on the chemical composition of lavender oils has been investigated. Liu et al. [1] reported that while linalool and linalyl acetate contents increased with MAE, in contrast to hydro-distillation, lavandulyl acetate and α-terpineol contents decreased. Rashed et al. [3] examined the efficacy of ultrasound-assisted microwave extraction in Chinese lavender oil. They discovered that ultrasound increased the efficiency of the extraction by rupturing plant tissue with acoustic cavitation, reporting major compounds such as linalool (32.9%), linalyl acetate (27%), and lavandulyl acetate (15.6%). Furthermore, Perino-Issartier et al. [4] and Filly et al. [15] studied the chemical makeup of lavender oils extracted using hydro-distillation, steam distillation, and microwave treatment. Both studies revealed that the concentration of linalyl acetate was greater in lavender oil extracted using solvent-free microwave methods and steam distillation, as opposed to hydro-distillation. Researchers have suggested that, during hydro-distillation in the presence of water, linalyl acetate breaks down into linalool and eventually 4-terpineol, unlike other methods that involve indirect contact with water. It has also been reported that the degradation of linalool to 4-terpineol in aqueous solutions at higher temperatures is higher [15]. It is interesting to note that linalyl acetate (36.19%) and linalool (28.29%) were higher in the MAE oil in this study, whereas 4-terpineol (6.26%) was higher in the SCDE oil. While both methods do not involve the direct interaction of water, the conversion of linalool to 4-terpineol may have been influenced by the high-pressure processing. Furthermore, the concentrations of these major compounds were very similar to what we found in the literature, despite the environmental drawbacks (arid and salty soil) of Konya Province in Türkiye. In line with previous research, this investigation shows that the chemical composition of essential oils is greatly influenced by both environmental conditions and extraction techniques. Additionally, the results indicate that an essential oil extraction process can be selected to achieve the desired chemical profile. For instance, this could be especially significant if the essential oil’s chemical constituents have been associated with specific bioactivity. Table 3 demonstrates the medicinal properties of major compounds found in lavender oil.

The impact of the extraction method on the yields of lavender oil is illustrated in Table 4. The yield values were not significantly impacted by the extraction method (*p* > 0.05); however, supercritical carbon dioxide extraction produced a higher yield. The results show that the yield values for MAE and SCDE were 2.85% and 2.94%, respectively, which are also within the range of previous research [6,12,19].

### 3.2. Assessment of Antioxidant Activity

The FRAP and DPPH free-radical scavenging activity assays were used in this investigation to quantify the antioxidant activity of lavender oils. Antioxidant tests involve various reaction pathways, such as the antioxidant donating an electron or transferring a hydrogen atom [31]. In the DPPH test, the stable radical 2,2-diphenyl-1-picrylhydrazyl, or DPPH, has the ability to absorb both an electron and a hydrogen atom. The extract solution turns yellow when antioxidants react with purple-colored DPPH. The decrease in absorbance at 517 nm is directly correlated with the presence of the decreased DPPH radical [31]. In the FRAP assay, the ferric iron is reduced to ferrous iron by an electron donation. Ferrous-tripyridyl-S-triazine (TPTZ) is an iron-binding ligand that has a dark blue color at 593 nm. Higher levels of antioxidants cause more ferric iron to convert to ferrous iron, which intensifies the dark blue tone of the color [31]. The antioxidant assay results are displayed in Table 4. In both tests, the antioxidant capacity of the MAE lavender oil was found to be greater than that of the SCDE oil. Additionally, DPPH antioxidant activity was statistically significantly (*p* ≤ 0.05) greater in MAE lavender oil compared with SCDE lavender oil, with IC_50_ values of 72.99 mg/mL and 80.84 mg/mL, respectively. Lower IC_50_ values indicate higher antioxidant activity. In addition, the results of the FRAP experiment for the MAE and SCDE lavender oils were 1.31 mM Fe^2+^/g and 1.14 mM Fe^2+^/g, respectively. As the literature on the antioxidant qualities of lavender oil is not well documented, the results of this study serve as a comparative reference for future research.

It has previously been shown that heavier, non-volatile molecular-weight compounds with lower antioxidant activity can be extracted by using high-pressure levels in supercritical carbon dioxide extraction [32]. This suggests that the extraction conditions of SCDE in this study may lead to the extraction of less volatile compounds with lower antioxidant activity. Additionally, the extraction of more components using the SCDE method also supports these findings. In another study, the DPPH radical scavenging activity of lavender oils was examined using supercritical carbon dioxide extraction and hexane extraction [6]. In this case, hexane extraction caused the extraction of heavier, non-volatile compounds with lower DPPH radical scavenging activity. Consequently, it can be said that different compounds with varied bioactivity can be obtained depending on the temperature, applied pressure, and type of solvent employed in the extraction procedures.

Furthermore, as previously mentioned, terpenes, oxygenated terpenes, and other bioactive chemicals have been linked to the antioxidant activity of essential oils [9,10]. It was also discovered that the presence and placement of a hydroxyl group inside these molecules affect the antioxidant activity of essential oils [33]. Two major monoterpenoids found in the lavender oils, linalool and linalyl acetate, were found in higher concentrations in the MAE lavender oil compared to the SCDE lavender oil, which may have contributed to the higher antioxidant activity of the MAE oil.

### 3.3. Evaluation of Antibacterial Activity Through In Vitro Testing

The antibacterial properties of the extracted oils were evaluated using two different assays consisting of the agar disc diffusion test and the broth dilution test. In these tests, the antibacterial activity of the oils was assessed against a total of nine bacterial strains, including six Gram-positive and three Gram-negative rods and cocci, which are major foodborne pathogens (excluding *E. coli* ATCC 25922) associated with foodborne illnesses. The results of the antibacterial tests are presented in Table 5.

The extracted oils demonstrated antibacterial activity against the pathogens tested in both the agar disc diffusion and broth dilution methods, showing varying degrees of inhibition. It has been reported that there is a relationship between the volatile component profile of the oils and their antibacterial activity [34]. Previous studies have indicated that volatile components such as linalool, linalyl acetate, and borneol in lavender oil are responsible for its bacteriostatic properties against various Gram-positive and Gram-negative bacteria [5,6]. Indeed, linalool and linalyl acetate were identified as the dominant volatile components in the oil samples (Table 2).

**Figure 4:**
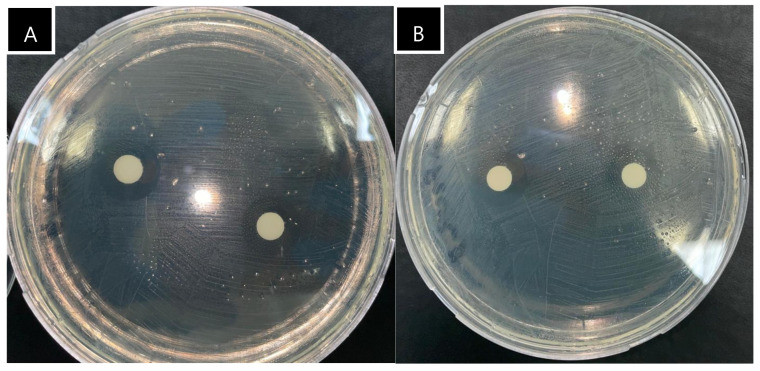
Inhibition zones of MAE (**A**) and SCDE (**B**) lavender oils against Gram-positive pathogenic bacteria *S. aureus*.

According to the agar disc diffusion results, it was observed that different extraction methods affected the antibacterial activity against Gram-positive *E. faecalis* ATCC 29212 and *L. monocytogenes* ATCC 19115. The antibacterial activity against both bacteria was found to be higher in the MAE lavender oil compared with the SCDE lavender oil (*p* ≤ 0.05). This difference could be attributed to the higher quantities of endo-borneol, alpha-terpineol, linalyl acetate, and lavandulyl acetate in the MAE lavender oil compared with those in the SCDE oil (Table 2). Endo-borneol and alpha-terpineol have been reported to possess antilisterial effects [35]. Although endo-borneol and alpha-terpineol were not identified as major components in the oil samples, the minor components in the chemical composition were noted to significantly contribute to the antibacterial activity [13]. Additionally, linalool and linalyl acetate have been reported as significant components in the inhibition of *E. faecalis* strains [36]. On the other hand, no significant differences in antibacterial activity were observed between the extraction methods for the other tested bacteria (*p* > 0.05), as evidenced by the inhibition zones of *S. aureus* illustrated in Figure 4.

Regardless of the extraction methods, it was observed that Gram-positive bacteria (excluding *E. faecalis*) were more sensitive to the extracted oils compared with Gram-negative bacteria, as evidenced by the inhibition zones (Table 5). This can be attributed to the hydrophilic outer membrane of Gram-negative bacteria, which limits the penetration of hydrophobic components such as oils, thereby reducing antibacterial activity [37]. Danh et al. [6] reported that oils extracted using SCDE from lavender in Victoria (Australia) exhibited inhibition zones of 28.1 mm, 11.4 mm, and 28.1 mm against *S. aureus* (ATCC 26923), *E. faecalis* (ATCC 19433), and *E. coli* (ATCC 25922) strains, respectively. Another study showed that oils extracted using SCDE from lavender in Mersin (Türkiye) displayed inhibition zones of 31.3 mm, 37.0 mm, and 24.7 mm against *B. subtilis*, *S. aureus*, and *E. coli*, respectively [12]. On the other hand, Sadani and Shakeri [38] observed that oils extracted using MAE from lavender collected from Jahan Nama Mountain (Gorgan, Golestan Province) exhibited inhibition zones of 14 mm, 29 mm, 17 mm, and 32 mm against *S.* Enteritidis PTCC 1639, *B. cereus* ATCC 1247, *E. coli* PTCC 1399, and *S. aureus*, respectively. In this study, lavender oils extracted using SCDE from plants grown in arid soils exhibited inhibition zones of 11.5 mm, 11.5 mm, and 9.5 mm against *B. subtilis* PY79, *S. aureus* ATCC 9144, and *E. coli* ATCC 25922, respectively. For the MAE lavender oil, the inhibition zones were 11.0 mm, 9.0 mm, and 8.0 mm, respectively. In another study, researchers discovered that when compared with hydro-distillation, lavender oil extracted using the microwave technique had greater antimicrobial efficacy against *B. subtilis* and *Actinomyces viscous* [1]. These results suggest that the variety of the plant, geographic conditions, and extraction methods significantly influence antibacterial properties. Indeed, different extraction methods impact the chemical composition of lavender oil, particularly the concentrations of linalyl acetate and linalool [15]. It has also been reported that drought stress reduces the biosynthesis of antibacterial components such as linalool in lavender [39]. Therefore, the antibacterial activities of lavender oils extracted from drought-affected regions might be lower than those from non-drought-stressed areas.

It is well-established that the broth dilution test provides more sensitive results compared with the agar disc diffusion technique [5]. While differences in extraction methods were significant for *E. faecalis* ATCC 29212 in both analysis methods, the impact of the extraction method was particularly notable in the inhibition of *B. cereus* NRRL B3711 and *B. subtilis* PY79 in the broth dilution test (*p* ≤ 0.05). The broth dilution test also confirmed that Gram-positive bacteria were more sensitive to the extracted lavender oils. According to the broth dilution test results, the MAE lavender oil demonstrated twice the antibacterial activity of the SCDE lavender oil (Table 5). Specifically, the MIC values required to inhibit *B. cereus* NRRL B3711, *B. subtilis* PY79, and *E. faecalis* ATCC 29212 were half as high for the MAE lavender oil compared with the SCDE lavender oil. The variations in the quantitative composition (Table 2) of oils obtained via different extraction methods are supposed to have an impact on the MIC values. Gram-positive bacilli such as *Bacillus* species have been reported to be sensitive to lavender oil, particularly due to its linalool and linalyl acetate contents [40]. These components exhibit antibacterial effects by damaging bacterial cell membranes and affecting enzyme functions, thereby disrupting metabolic processes. Consequently, the relatively higher linalool and linalyl acetate contents in the MAE lavender oil compared with the SCDE lavender oil may have resulted in lower MIC values and higher antibacterial activity in the MAE oil.

In the broth dilution test, Rota et al. [5] found that oils obtained via hydro-distillation from lavender grown in the dry continental climate of northeastern Spain (Aragon) exhibited no antibacterial activity against *S.* Enteritidis CECT 4155, *S.* Typhimurium CECT 443, and *E. coli* O157:H7 CECT 4267. However, in this study, antibacterial activity against these species was observed, with MIC values ranging from 60 to 120 mg/mL. The difference between the MAE and SCDE methods and hydro-distillation may be caused by the presence and concentration of bioactive chemicals linked to antibacterial properties, as indicated by prior studies of the essential oils of other aromatic and medicinal plants, such as thyme and black thyme [9], sweet basil leaves [41], and coriander seed [42]. Additionally, oils obtained via microwave-assisted extraction from lavender grown in non-arid climates showed an MIC value of 0.25 mg/mL against *S. aureus* CCTCC AB 91093 and *B. subtilis* CCTCC AB 90008. These findings are consistent with the agar disc diffusion results, indicating that lavender cultivation under drought conditions reduced antibacterial activity. In conclusion, the data suggest that the extraction method significantly affects the bioactivity of the oils, with MAE proving to be superior to SDCE in terms of the antibacterial activity of oils extracted from lavender grown in arid regions.

## 4. Conclusions

This study provided a comparative analysis of essential oils of lavender cultivated in a drought-affected region of Türkiye. Lavender essential oils were extracted from plants using MAE and SCDE methods and analyzed to determine their chemical composition, antioxidant capacity, and antibacterial properties. The GC-MS analysis revealed significant differences in the chemical profiles of the oils obtained through the two extraction methods. The MAE oil showed higher concentrations of key bioactive compounds, such as linalyl acetate (36.19%) and linalool (28.29%), when compared with the SCDE oil, which exhibited values of 28.72% and 27.48%, respectively. Additionally, the MAE lavender oil not only contained higher levels of endo-borneol, it also included alpha-terpineol. These differences are crucial, as these compounds are known for their potent antibacterial and antioxidant properties. The antioxidant assays (DPPH and FRAP) demonstrated that the MAE oil had superior antioxidant activities. The DPPH assay showed an IC_50_ value of 72.99 mg/mL for the MAE lavender oil, which was significantly lower (indicating higher antioxidant activity) than the IC_50_ value of 80.84 mg/mL for the SCDE oil. Similarly, the FRAP assay results were 1.31 mM Fe^2+^/g for MAE and 1.14 mM Fe^2+^/g for SCDE, further supporting the superior antioxidant potential of the MAE oil. The antibacterial efficacy of the oils was evaluated against nine bacterial strains using both agar disc diffusion and broth dilution methods. The MAE oil exhibited higher antibacterial activity, with lower MIC values for key pathogens. The MIC values for *B. cereus* NRRL B3711, *B. subtilis* PY79, and *E. faecalis* ATCC 29212 were 30.0 mg/mL, 60.0 mg/mL, and 30.0 mg/mL, respectively, for the MAE oil, compared with 60.0 mg/mL, 120.0 mg/mL, and 60.0 mg/mL for the SCDE oil. Overall, the results highlight the significant impact of the extraction method on the bioactivity of lavender essential oils. The MAE method exhibited better antioxidant and antimicrobial activities compared with SCDE. Additionally, the research findings indicated that lavender flowers, which contain chemicals with high bioactivity, such as linalool and linalyl acetate, can be grown even in places characterized by high environmental stress conditions.

## Figures and Tables

**Table 1:** GC-MS conditions used for the determination of the chemical composition of *Lavandula angustifolia* essential oils.

Instrument	GCMS-QP2020 equipped with Waters 2695 pumps
Type of column	Rxi-5Sil MS column (5% diphenyl-95% dimethylpolysiloxane 30 m × 0.25 mm I.D., df = 0.25 m) (RESTEK GC Columns, USA)
Injector and detector temperatures	250 °C
Temperature program	2 min at 40 °C, 4 °C increments per minute up to 240 °C, and hold for 53 min
Injection volume	1 µL (diluted with n-hexane 1:10, *v/v*)
Flow rate	43.4 cm/s
Carrier gas	Helium
Injection	Split mode (1:25)
Ionization voltage	70 electron volts (eV)
Mass-to-charge ratio	40–400 amu (atomic mass units)

**Table 2:** Chemical characteristics of *Lavandula angustifolia* essential oils using microwave and supercritical CO_2_ extraction.

No.	Compounds	RI ^A^	RI ^B^	Relative Peak Area (%)	
				MAE ^A^	SCDE ^B^
1	Linalool oxide trans	18.607	18.634	1.35 ± 0.16 a	1.37 ± 0.22 a
2	Linalool oxide cis	19.262	19.287	0.93 ± 0.06 a	1.01 ± 0.24 a
3	Linalool	19.990	20.163	28.29 ± 0.95 a	27.48 ± 2.89 a
4	Lavandulol	22.419	22.497	1.07 ± 0.13 a	1.13 ± 0.22 a
5	Endo-borneol	22.913	22.993	1.35 ± 0.18 a	0.81 ± 0.00 b
6	4-terpineol	23.206	23.311	4.87 ± 0.16 a	6.26 ± 0.93 a
7	Alpha-terpineol	23.779	--	1.10 ± 0.16	--
8	3,7-octadiene-2,6-diol, 2,6-dimethyl	--	23.803	--	1.67 ± 0.13
9	Linalyl acetate	25.770	25.872	36.19 ± 0.71 a	28.72 ± 2.26 b
10	Lavandulyl acetate	26.900	26.961	6.24 ± 0.16 a	5.59 ± 0.05 b
11	3,7-dimethyl-1,5-octadiene-3,7-diol	28.765	28.858	0.21 ± 0.02 b	1.69 ± 0.07 a
12	1-hydroxylinalool	29.126	29.173	1.29 ± 0.67 a	2.02 ± 0.69 a
13	Trans-beta-caryophyllene	32.120	32.192	2.55 ± 0.19 a	3.84 ± 2.16 a
14	Farnesene <(E)-, beta->	32.932	32.959	1.03 ± 0.14 a	1.30 ± 0.04 a
15	(−)-Caryophyllene oxide	37.578	37.633	2.96 ± 0.38 a	4.51 ± 1.61 a
	Total peak area (%)			89.43%	87.40%

Values are expressed as means ± standard deviations (*n* = 3). RI ^A^: refractive index of MAE lavender oil. RI ^B^: refractive index of SCDE lavender oil. a,b: different letters represent statistically significant (*p* ≤ 0.05) differences between the treatments; main compounds (0.5% ≥) were reported. MAE: microwave-assisted extraction. SCDE: supercritical carbon dioxide extraction.

**Table 3:** Bioactivity of major compounds found in lavender essential oil.

Compounds	Bioactivity	Reference
Linalool	Induces cancer cell apoptosis via oxidative stress	[23]
	Strong antimicrobial activity against various pathogenic bacteria strains, including *Staphylococcus* spp., *Salmonella* spp., and *Pseudomonas* spp.	[24]
4-terpineol	Significant larvicidal and fumigant activities against *P. xylostella*	[25]
	Anticancer, anti-inflammatory, and antioxidant effects	[26]
Linalyl acetate	High peroxyl radical scavenging activity in both in vitro and in vivo studies	[27,28]
Lavandulyl acetate	Potential to increase vascular endothelial growth factor	[29]
(−)-Caryophyllene oxide	In vivo analgesic and anti-inflammatory properties; anticancer, immunomodulatory, antimicrobial, and antioxidant activities	[30]

**Table 4:** Yield and antioxidant activity of *Lavandula angustifolia* essential oils using microwave and supercritical CO_2_ extraction.

Extraction Method	Yield (%)	DPPH (IC_50_, mg/mL)	FRAP (mM Fe^2+^/g)
MAE	2.85 ± 0.25 a	72.99 ± 1.02 a	1.31 ± 0.15 a
SCDE	2.94 ± 0.06 a	80.84 ± 0.56 b	1.14 ± 0.14 a

Values are expressed as means ± standard deviations (*n* = 3). a,b: different letters represent statistically significant (*p* ≤ 0.05) differences between the treatments. MAE: microwave-assisted extraction. SCDE: supercritical carbon dioxide extraction. DPPH: 2,2-diphenyl-1-picrylhydrazyl. FRAP: ferric-reducing antioxidant power.

**Table 5:** The results of agar disc diffusion (mm) and broth dilution (mg/mL) assays for *Lavandula angustifolia* essential oils obtained using microwave and supercritical CO_2_ extraction.

Bacterial Species	MAE		SCDE	
	IZ (mm) *	MIC (mg/mL) *	IZ (mm) *	MIC (mg/mL) *
*Bacillus cereus* NRRL B3711	9.0 ± 0.2 a	30.0 ± 0.0 A	8.5 ± 0.5 a	60 ± 0.0 B
*Bacillus subtilis* PY79	11.5 ± 0.2 a	60.0 ± 0.0 A	11.0 ± 0.5 a	120 ± 0.0 B
*Enterococcus faecalis* ATCC 29212	6.0 ± 0.0 a	30.0 ± 0.0 A	3.5 ± 0.1 b	60 ± 0.0 B
*Listeria monocytogenes* ATCC 19115	9.0 ± 0.5 a	120.0 ± 0.0 A	6.0 ± 0.0 b	120 ± 0.0 A
*Staphylococcus aureus* ATCC 9144	11.5 ± 0.2 a	120.0 ± 0.0 A	9.0 ± 0.0 a	120 ± 0.0 A
*Staphylococcus epidermidis* ATCC 12228	9.5 ± 0.1 a	60.0 ± 0.0 A	8.0 ± 0.0 a	60 ± 0.0 A
*Escherichia coli* ATCC 25922	9.5 ± 0.1 a	60.0 ± 0.0 A	8.0 ± 0.0 a	60 ± 0.0 A
*Salmonella* Enteritidis ATCC 13076	7.5 ± 0.1 a	60.0 ± 0.0 A	5.5 ± 0.5 a	60 ± 0.0 A
*Salmonella* Typhimurium ATCC 14028	5.0 ± 0.1 a	120.0 ± 0.0 A	5.0 ± 0.0 a	120 ± 0.0 A

* Values are expressed as means ± standard deviations (*n* = 3). A,B,a,b: different letters represent statistically significant (*p* ≤ 0.05) differences between the treatments. MAE: microwave-assisted extraction. SCDE: supercritical carbon dioxide extraction. IZ: inhibition zone. MIC: minimum inhibitory concentration.

## Data Availability

The original contributions presented in this study are incorporated in the article; for further information, contact the corresponding author.
